# Cloze, Frequency, Surprisal, or Plausibility? A Comparative Analysis of Predictors for Local Ambiguity Resolution

**DOI:** 10.1111/cogs.70208

**Published:** 2026-04-30

**Authors:** Markéta Ceháková, Jan Chromý

**Affiliations:** ^1^ Institute of Czech Language and Theory of Communication, Faculty of Arts Charles University

**Keywords:** Reanalysis, Surprisal, Plausibility, Structural bias, Garden‐path sentences

## Abstract

This study investigated the cognitive mechanisms underlying the processing of garden‐path sentences by examining the influence of verb/structural bias, cloze probability, surprisal, and plausibility. Using self‐paced reading with yes/no comprehension questions, we analyzed a structurally diverse set of 11 types of ambiguous and unambiguous sentences. Our results revealed that cloze probability was the most robust predictor of processing difficulty, significantly influencing both reaction times and response accuracy. Specifically, the likelihood of a misanalysis, as indexed by cloze scores, predicted the persistence of incorrect interpretations and reanalysis difficulty. In contrast, verb bias, surprisal, and plausibility exerted weaker or inconsistent effects, with only plausibility showing a limited interaction in the accuracy data. These findings suggest that comprehenders rely heavily on contextual cues when interpreting syntactically ambiguous input, and that reanalysis success depends not solely on structural preferences or lexical predictability but on the overall likelihood of the initial misanalysis and of the intended interpretation.

## 1 Introduction

Garden‐path sentences like (1) belong to the most extensively studied phenomena in psycholinguistic research. Their temporarily ambiguous nature makes them a valuable tool for exploring various aspects of sentence processing such as the good‐enough properties of linguistic representations (Christianson, Hollingworth, Halliwell, & Ferreira, 2001; Huang & Ferreira, 2021; Slattery, Sturt, Christianson, Yoshida, & Ferreira, 2013), processes of re‐reading and reanalysis (Christianson, Dempsey, Tsiola, Deshaies, & Kim, 2024; Paape & Vasishth, 2022), information retrieval and interference (Fujita & Vasishth, 2024; Martin & McElree, 2018), and the roles of predictability and surprisal (Huang et al., 2024; van Schijndel & Linzen, 2021).

(1) While Anna dressed the baby played in the crib.

In sentence (1), the region *dressed the baby* is locally ambiguous. It can be interpreted as a combination of a transitive verb followed by its object (*While Anna dressed the baby…*) or, alternatively, as a reflexive verb and a subject of a second clause (*While Anna dressed herself, the baby played…*). Readers tend to adopt the transitive interpretation at first, but this is later disconfirmed by the disambiguating verb *played*, which lacks a subject if the original parse is maintained. As a result, readers are forced to revise their initial analysis and reinterpret the sentence.

This reanalysis process has been shown to impact various behavioral measures. The disambiguating region typically elicits elevated reaction times (RTs) in self‐paced reading and longer eye‐movement metrics (Christianson et al., 2001; Christianson, Luke, Hussey, & Wochna, 2017; Christianson et al., 2024; Ferreira & Clifton, 1986; Ferreira & Henderson, 1991; Slattery et al., 2013), as well as electrophysiological responses such as the P600 component (Gouvea, Phillips, Kazanina, & Poeppel, 2010; Osterhout, Holcomb, & Swinney, 1994; Qian, Garnsey, & Christianson, 2018). It was also shown to have significant effects in experiments using transcranial direct current stimulation targeting brain regions related to executive control (Hussey, Ward, Christianson, & Kramer, 2015). Processing difficulties also appear in off‐line measures, including response accuracy or picture selection (Christianson et al., 2001; Malyutina & den Ouden, 2016). For instance, readers often fail to suppress the initial misinterpretation, even after attempting reanalysis, leading to errors in responses to comprehension questions or in sentence paraphrasing (Christianson et al., 2017; Huang & Ferreira, 2021; Patson, Darowski, Moon, & Ferreira, 2009).

The size of the processing slow‐down (Huang et al., 2024) and the success of inhibiting the initial misanalysis (Christianson et al., 2001; Christianson & Luke, 2011; Christianson et al., 2017; Fujita & Cunnings, 2020) vary substantially across different garden‐path sentences. As Huang et al. (2024) demonstrate, there is considerable variation in the processing slow‐down on the disambiguating region not only between different types of garden‐path sentences (i.e., sentences with a different syntactic structure) but also between different sentences of the same type (i.e., sentences with the same syntactic structure but different content).

Similar variation also exists in response accuracy. For example, when participants answer questions targeting the initial misanalysis (such as *Did Anna dress the baby?* for sentence (1)), accuracy rates range from around 80%—as in sentence (2) (Christianson & Luke, 2011)—to as low as 20% for sentences like (3) (Christianson et al., 2017). This suggests that some garden‐path structures are more prone to misanalysis and/or more resistant to attempts at inhibiting this misanalysis.

(2) The publisher called up the editor and the author refused to change the book's ending.
(3) The player tossed the ball interfered with the other team.

Moreover, final sentence representations of garden‐path sentences can be disrupted even beyond the scope of the initial misanalysis of the ambiguous region (Chromý, 2022; Ceháková & Chromý, 2023). Sometimes, the ambiguous region does not get analyzed correctly at all (Christianson, Williams, Zacks, & Ferreira, 2006; Ceháková & Chromý, 2025; Hussey et al., 2015; Malyutina & den Ouden, 2016). Various other parts of the sentence might get misanalyzed too, and the final representation may be fragmented, consisting of multiple mutually exclusive treelets. The nature and extent of this disruption appear to vary by the type of garden‐path structure. For example, in sentences like (3), even the disambiguating verb (*interfered*) is frequently misanalyzed—an issue not typically observed in sentences like (1) (Ceháková & Chromý, 2025).

Further evidence comes from Fujita (2024), who shows that in certain very complex garden‐path structures, such as (4), the intended interpretation of the ambiguous region often does not emerge at all. In contrast, slightly less complex sentences—like (5)—are more likely to be analyzed as intended.

(4) While the friends telephoned the woman that Rebecca visited dropped a wine bottle and cut herself on a piece of broken glass.
(5) While the friends telephoned the woman that visited Rebecca dropped a wine bottle and cut herself on a piece of broken glass.

What causes the differences in processing and resulting representations of garden‐path sentences remains a long‐standing question. Although previous research has linked these differences to a range of linguistic properties, no definitive answers have been found. The variation in response accuracy across different garden‐path structures suggests that the syntactic structure of these sentences may play a role. For example, a series of experiments by Dempsey et al. (2020, 2024) focused on syntactic adaptation show that people track structural frequencies when processing linguistic input and rely on them in order to disambiguate garden‐path sentences. At the same time, inter‐item variability documented by Huang et al. (2024) points toward the influence of nonsyntactic factors, such as plausibility or frequency.

In the following section, we review several studies focusing on factors that may influence the processing of garden‐path sentences, and their relation to: (a) the likelihood that comprehenders experience a garden‐path effect (i.e., under what conditions do comprehenders get garden‐pathed), and (b) the difficulty of recovering from the initial misanalysis (i.e., what makes reanalysis more or less successful). We compare their findings to a recent study by Huang et al. (2024), which tested these predictors with a large participant and stimuli sample and did not find consistent evidence for their impact on processing of local ambiguities.

One of the earliest factors linked to the presence of the garden‐path effect was the *syntactic structure*. According to Frazier & Fodor (1978) and Frazier & Rayner (1982), misanalysis arises because the parser tends to favor the simplest possible syntactic structure (i.e., the one requiring the fewest nodes). When the correct analysis of a region is syntactically more complex than an incorrect one, as in sentence (1), misanalyses arise. Conversely, when the correct analysis is syntactically simple, the garden‐path effect is reduced or may not occur at all. Similar findings were reported by Ferreira & Clifton (1986) or Ferreira & Henderson (1991).

As for the ease of recovery from the initial misanalysis, some studies show that the key factor is not the number or type of syntactic operations required to fix the misanalysis, but rather the difficulty of diagnosing what went wrong in the initial parse (Fodor & Inoue, 1994, 1998). In particular, recovery is facilitated when the disambiguating region provides informative cues about how to reinterpret the sentence—such as morphosyntactic features that help identify the correct attachment site. In contrast, if the disambiguation merely signals an error without offering cues for reanalysis, recovery becomes more difficult (Fodor & Inoue, 2000). The informativeness of disambiguation might also depend on its semantic and pragmatic properties (Martin & McElree, 2018).

Another explanation for the presence and magnitude of the garden‐path effect relates to *frequency/structural bias* (Garnsey, Pearlmutter, Myers, & Lotocky, 1997; Trueswell, Tanenhaus, & Kello, 1993; Trueswell, 1996). For example, RTs at the disambiguating region are longer in sentences like (6), where the ambiguous verb frequently takes an NP complement (and comprehenders are thus more prone to misanalyzing it) than in sentences like (7), where the verb strongly prefers a sentential complement. In other words, the garden‐path effect is less likely to occur when the misanalysis does not align with the verb's bias, even if it is structurally simpler.

(6) The student forgot the solution was in the back of the book.
(7) The student hoped the solution was in the back of the book.

To our knowledge, no studies have yet examined frequency effects in relation to response accuracy, which would offer insights into reanalysis success and the nature of the resulting representation.

Another factor that may influence garden‐path sentence processing is *plausibility*. Several studies have shown that the garden‐path effect diminishes or disappears when the potential misanalysis of the ambiguous region describes an implausible scenario. For example, as shown by Trueswell, Tanenhaus, and Garnsey (1994), sentences like (9)—in which the misanalysis (i.e., the room searched something) is implausible—do not produce elevated RTs at the disambiguating region (*by the police*). In contrast, sentences like (8), where the misanalysis is more plausible, elicit longer reading times.

(8) The thief searched by the police was quite unpleasant.
(9) The room searched by the police was quite unpleasant.

A similar effect was also documented in a graded, item‐by‐item analysis by Nakamura & Arai (2016) and by Qian et al. (2018), who show that more plausible initial misanalyses lead to greater processing difficulty, as reflected in longer reaction times (RTs) at the disambiguating region. On the other hand, Garnsey et al. (1997) showed that plausibility influenced sentence processing only when the ambiguous region was equally biased toward the two potential interpretations (the initial misanalysis or the intended analysis). In other words, plausibility interacted with verb bias: when the ambiguous region strongly favored one interpretation, plausibility of the continuation did not significantly affect disambiguation difficulty.

Plausibility of the initial misanalysis also influences the success of sentence reanalysis. For example, in sentences like (10), where the potential misanalysis leads to an unlikely scenario (e.g., hunting planes), participants make fewer incorrect responses to the question targeting the potential misanalysis (*Did the man hunt the deer/plane?*) than their more plausible counterparts (11) and (12) (Malyutina & den Ouden, 2016).

(10) While the man hunted the plane flew over the woods.
(11) While the man hunted the deer ran into the woods.
(12) While the man hunted the deer paced in the zoo.

Interestingly, (12) also elicits fewer incorrect responses than (11). Both of these sentences tend to be misanalyzed, but the misanalysis in (12) is contradicted by the rest of the sentence (since we do not typically hunt animals in a zoo). This makes reanalysis easier and reduces the likelihood that the initial misanalysis will persist or blend with the intended analysis (Christianson et al., 2001; Malyutina & den Ouden, 2016).

However, the evidence for the influence of misanalysis plausibility on the final representation of the sentence is mixed. For example, a study by Roberts & Felser (2011) shows that the implausibility of the initial misanalysis improved reanalysis success (as measured by higher response accuracy), but only for a specific type of garden‐path sentences. Similarly, Nakamura & Arai (2016) observed effects of plausibility on response accuracy only in sentences with longer ambiguous regions: when the ambiguous region was short, participants responded correctly regardless of plausibility, but for longer regions, plausible misanalyses led to more errors.

Other conflicting results come from Qian et al. (2018), who show that response accuracy to questions targeting the initial misanalysis was not reliably influenced either by the plausibility of the initial misanalysis itself (e.g., a man hunted a deer) or by the plausibility of the misanalysis in the context of the rest of the sentence (e.g., a man hunted a deer that ran into the woods). Effects were only found for certain sentence types and only in some of the experiments. Qian et al. (2018) did, however, find that when the entire event described by the garden‐path sentence (while the man hunted the deer ran into the woods) was considered more plausible, the response accuracy was lower. However, this could have been caused by the experimental design, since the question used for the plausibility judgment (“How likely is it that the man hunted the deer?”) was basically a differently phrased misanalysis question (“Did the man hunt the deer?”), which could potentially lead to a high correlation between those two measures.

Recently, several studies have examined whether the presence and magnitude of processing slow‐down in garden‐path sentences can be explained by *surprisal* of the disambiguating region (Huang et al., 2024; van Schijndel & Linzen, 2021). Once again, the results are mixed. While large‐language‐model (LLM) surprisal estimates at the critical region can predict the presence and direction of the garden‐path effect, they greatly underestimate its magnitude. Moreover, surprisal estimates failed to capture differences in processing difficulty between different types of garden‐path sentences or between items within the same structural type. These results suggest that other mechanisms—such as reanalysis—likely contribute to processing difficulty at the point of disambiguation.

To our knowledge, no studies have yet investigated the effects of next‐word surprisal on response accuracy. For example, while Huang et al. (2024) collected response accuracy data, they did not analyze them in relation to surprisal values.

The summary above is, of course, not definitive. Several additional factors have been hypothesized to influence processing speed and response accuracy in garden‐path sentences. Among them is, for example, *extrasentential context* (i.e., when preceding sentences pragmatically bias the locally ambiguous sentence toward a particular interpretation), which can either strengthen or weaken the garden‐path effects described above (Altmann, Garnham, & Dennis, 1992; Christianson & Luke, 2011). The ease of recovery from a garden‐path might also be related to factors of a more *cognitive* nature. It has been documented that the success of reinterpreting and inhibiting the initial misanalysis depends on how much time has passed since this analysis was committed to memory: the longer the delay (i.e., the longer the segment between the ambiguous and the disambiguating region), the more difficult it is to abandon the misanalysis and find the correct interpretation (Christianson et al., 2001; Ferreira & Henderson, 1991; Tabor & Hutchins, 2004). Other relevant factors may include the amount of cognitive load placed on memory during parsing, for example, due to retrieval interference (Martin & McElree, 2018; Van Dyke & Lewis, 2003) or the need to maintain long‐distance dependencies (Liu, 2008).

The brief overview presented above captures the general state of research on recovery from garden‐path sentences while highlighting its limitations. Although these earlier studies were detailed—often combining multiple experimental methods with carefully prepared and normed stimulus sets—they sometimes yielded conflicting findings. Effects observed in one study were not always replicated in others, and occasionally, even opposite effects were reported (cf. sections on frequency/structural bias and plausibility).

Recently, Huang et al. (2024) ran a large‐scale self‐paced reading study, in which they investigated the role of several potential predictors (namely, the bias of the structure toward the ultimately correct analysis as measured by cloze task and by corpus frequencies, plausibility of the misanalysis, and surprisal of the disambiguation) on the speed of processing of three different types of garden‐path structures (as well as several other structures frequently used in psycholinguistics). This study worked with an impressive participant sample (*n* = 2000) as well as a diverse, carefully constructed set of stimuli (the three types of garden‐path sentences were each represented by 24 items). The stimuli were distributed in such a way that each item was seen by between 220 and 440 participants.

Interestingly, their study, despite its power, failed to replicate most of the effects observed in the previously mentioned papers. They documented clear differences in RTs between the three types of garden‐path structures used in the experiment, but also a diverse range of RTs within each type. However, this variety could not be reliably explained by the four linguistic variables mentioned above. Namely, they found no significant effects of the plausibility of the initial misanalysis on RTs for the spillover region. They found a strong effect of the cloze‐based verb bias, but only for one type out of the three garden‐path structures (the same one that was used by Garnsey et al. 1997). The corpus‐based verb bias also showed strong effects for one of the garden‐path types, but this effect went in the opposite direction than expected (i.e., the more frequent the correct analysis was, the bigger the garden‐path effect). Lastly, surprisal also showed effects only for one of the sentence types.

It thus appears that even this high‐powered, large‐scale replication provides further evidence of the inconsistency of the effects in question. Several factors may contribute to this variability.

First, a majority of the studies investigating factors that might influence sentence processing and its outcomes work with only a single type of garden‐path structure (though the specific type varies across studies). However, studies which *did* include multiple types of structures (e.g., Huang et al., 2024; Nakamura & Arai, 2016; Roberts & Felser, 2011) often documented substantial differences in how these structures were processed. In several cases, variables of interest (such as plausibility or structural bias) showed significant effects for one type of structure but not for another. This variation, often unaccounted for, might explain some of the inconsistencies observed in the above‐mentioned studies.

In the present study, we work with a diverse set of garden‐path sentences with various syntactic structures. These materials produce a wide range of garden‐path effects in both response accuracy and RTs, as well as variation in acceptability ratings. Employing a broad set of structures is important for two reasons: (a) if any predictor can explain the presence and magnitude of garden‐path effects *in general*—as is often implicitly assumed—then it should do so across constructions rather than within one or two of them; (b) a wider range of garden‐path effects increases the sensitivity of the analysis by making any systematic influence of the predictors easier to detect.

Second, studies differ substantially in how the individual predictors (such as plausibility or verb bias) are operationalized. Some treat these variables as binary and/or usually based on extreme values (e.g., very plausible/very implausible as in Garnsey et al., 1997; Nakamura & Arai, 2016)), while others use continuous measures in which the difference between the more plausible and less plausible items is not as stark (e.g., Huang et al., 2024; Qian et al., 2018). This might cause researchers to potentially miss out on more fine‐grained differences or lead to different outcomes based on their choice of materials. In our study, we systematically treat all predictors as continuous variables, allowing us to compare them directly and to capture more subtle variation in their influence on processing.

Finally, research on recovery from misanalysis has tended to focus mainly on the properties of the initial misanalysis of the ambiguous region, while often neglecting the role of other parts of the sentence—especially the disambiguating region and the correct analysis of the ambiguous region. However, these might also substantially influence the course of processing and its outcome. For example, at the point of disambiguation, comprehenders must decide where to attach the disambiguating region; its properties may thus be just as important for reanalysis as those of the ambiguous region. Sentence (13) then might be easier to analyze correctly than sentence (14), because the disambiguating verb *played* forms a more plausible, frequent, and predictable combination with *the baby* than the less typical verb *doomscrolled*. This difference might facilitate reanalysis due to higher informativity (Fodor & Inoue, 2000; Martin & McElree, 2018) and help inhibit the initial misanalysis more effectively. Our study extends the exploration beyond the level of the initial misanalysis and also assesses the properties of the disambiguating region and the correct interpretation of the ambiguous region as potential predictors of garden‐path difficulty.

(13) While Anna dressed the baby played in the crib.
(14) While Anna dressed the baby doomscrolled in the crib.

To sum up, the goal of the current study is to follow up on Huang et al. (2024) and explore the processing of garden‐path sentences and the factors that may influence it, while taking the above‐mentioned limitations into account. In the first part of the study, we explore a wide range of Czech garden‐path sentences, examining differences in RTs at the point of disambiguation and in response accuracy. In the second part, we explore the potential factors driving these differences. We consider properties of the misanalysis of the ambiguous region, the correct analysis of the ambiguous region, and the correct analysis of the disambiguating region. For each, we examine several variables previously explored in the literature and used by Huang et al. (2024) (plausibility, surprisal, cloze probability, and structural/verb bias), treating them as graded rather than binary variables.

Our study can thus be seen as an extension of Huang et al. (2024) to a new language, focusing on a wide range of Czech garden‐path structures, while also introducing new data useful for further analyses, namely, response accuracy and plausibility/structural bias/cloze probability/surprisal estimates for several relevant parts of the sentence (going beyond the initial misanalysis of the ambiguous region).

## 2 Current study

Our study provides a systematic investigation of how garden‐path sentences with diverse syntactic structures and varying degrees of subjective naturalness are processed and ultimately represented. We examine whether the observed variation can be accounted for by previously identified factors such as structural bias (measured by cloze task and corpus frequencies), surprisal, and plausibility. By clarifying the nature of these differences and the mechanisms that drive them, our findings contribute valuable insights into the process of syntactic reanalysis—shedding light on which factors affect the ease of recovery from misanalysis—and offer empirical support for competing theories of language processing.

### 2.1 Stimuli selection

The primary aim of this study is to investigate the role of various predictors in local ambiguity resolution. To this end, we employed a heterogeneous set of stimuli. Given that Czech is a morphologically rich language with relatively free word order and extensive case syncretism, it lends itself well to constructing a broad range of syntactically and semantically varied garden‐path structures.

Stimulus selection proceeded in two steps. First, we identified 11 distinct types of garden‐path sentences in Czech (see Appendix A for detailed description). For each type, we created eight items. All stimuli were carefully constructed to be relatively short, matched in length (eight words), and, as far as possible, matched in the length of the ambiguous region (with 1–3 intervening words between the ambiguous and disambiguating regions). Each garden‐path sentence had a closely matched unambiguous counterpart, differing only in the gender or number of the ambiguous noun (occasionally replaced with a close synonym) or in the conjunction used.

The second step involved assessing the heterogeneity of the stimulus set using a naturalness rating study (comprising 88 experimental items and 126 fillers, including 24 ungrammatical ones). The study was conducted online via the PC IbexFarm platform (Zehr & Schwarz, 2018), and included 164 Charles University students who participated for course credit.

Participants rated each sentence on a 100‐point slider scale, anchored with “completely unnatural” and “completely natural.” A “completely natural” sentence was defined as one that is comprehensible, error‐free, and easy to use or encounter in everyday language. A “completely unnatural” sentence was defined as its opposite.

Based on the results, we selected 66 sentences for the final study (six per each of the 11 garden‐path types). Outliers were identified within each sentence type, and up to two items per type were excluded. If fewer than two items were excluded based on the ambiguous condition, we additionally removed the lowest‐rated unambiguous items.

**Fig. 1:**
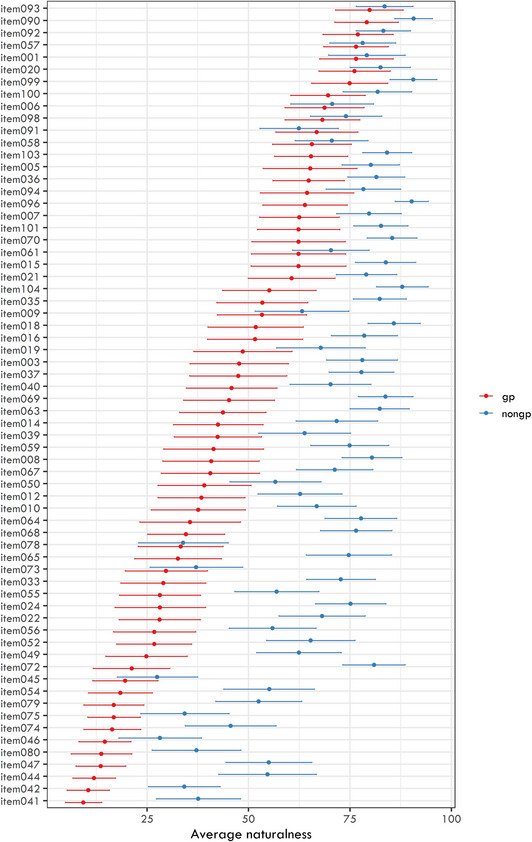
Average naturalness for individual items in two conditions (garden‐path and non‐garden‐path). Items are sorted in descending order based on their naturalness for garden‐path conditions.

The selected items varied in overall naturalness and in the contrast between ambiguous and unambiguous conditions. On average, unambiguous sentences were rated as more natural (mean = 68.89, *SD* = 16.63) than ambiguous ones (mean = 45.18, *SD* = 20.67). Item‐level results for both conditions are shown in Fig. 1.

Our goal for this study was to create a diverse set of garden‐path stimuli rather than to compare construction types directly. Some types show apparent tendencies (e.g., coordination ambiguities seem easier, while patient/beneficiary ambiguities are more difficult), but within‐type variability is high (see Appendix B for plots of accuracy and RT by item). A systematic analysis of structural differences in garden‐path difficulty would require a design with more items per construction type. In the present study, we focus instead on how these differences can be explained by continuous linguistic predictors such as surprisal, plausibility, and structural bias.

### 2.2 Sentence segments relevant for reanalysis

Unlike previous studies, our paper focuses on several regions of garden‐path sentences, not just the initial misanalysis. To fully and correctly (re)analyze a garden‐path sentence, comprehenders need to perform at least three steps, each related to different parts of the sentence. Specifically, they need to: (a) correctly attach the disambiguating region to its intended attachment site, (b) detach the ambiguous region from its initial position and inhibit this misanalysis, and (c) reanalyze (and reattach) the ambiguous region as intended (Fodor & Inoue, 1994). At least three different local strings are thus likely to be mentally represented during the reanalysis: the correct analysis of the disambiguating region (further called discor), the misanalysis of the ambiguous region (further called ambmis), and the correct analysis of the ambiguous region (further called ambcor). Example (15) illustrates the situation on a well‐known garden‐path structure cited earlier in the paper.

(15) While Anna dressed the baby played in the crib.
  1. discor: the baby played in the crib
  2. ambmis: Anna dressed the baby
  3. ambcor: Anna dressed herself

The properties of these three strings may significantly influence the ease and success of recovery from misanalysis. For example, the more frequent and plausible discor and ambcor are, the easier it might be to abandon ambmis. That is, if it is more likely that the baby is playing in the crib instead of being dressed, or that Anna is dressing herself rather than the baby, the comprehender may more readily arrive at the intended interpretation of the sentence and adhere to it while inhibiting the alternative. Conversely, when discor and ambcor are more surprising, additional processing effort may be required during reanalysis.

### 2.3 Predictors

Based on the previous studies, we identified several properties of the three regions described above that might influence the ease of recovery from the initial misanalysis. Specifically, we were interested in the role of cloze probability, verb/structural bias, surprisal, and plausibility. The following sections describe in more detail how each of these measures was obtained. All related data and code can be found here: https://tinyurl.com/d9mu7hpe.

#### 2.3.1 Cloze probability

We used a cloze task to estimate the degree to which each item is biased toward either the misanalysis of the ambiguous region (ambmis), or the correct analysis of this region (ambcor).

Data were collected from 115 participants, all of whom were Charles University students and participated for course credit. The experiment was administered online via the PC IbexFarm platform.

Participants were always presented with a sentence preamble, ending just before the disambiguating region, and were asked to complete the sentence in an open text field. For example, for sentence (16), participants saw the preamble *Luděk prohledal obchodnici před prodejnou…* (“Luděk searched the shopkeeper (acc./dat.sg.) in front of a shop…”). In this example, the noun *obchodnici* (“shopkeeper”) is ambiguous: it is initially interpreted as a direct object (acc.sg), but in the end must be reanalyzed as a beneficent/external possessor (dat.sg.).

(16) Luděk      prohleda‐l    obchodnic‐i     před
  Luděk‐nom.m.sg search‐3sg.m.pst shopkeeper‐dat.f.sg in front of
  prodejn‐ou   dodávk‐u   se  zbož‐ím.
  shop‐inst.f.sg van‐acc.f.sg with goods‐3sg.m.pst
  “Luděk searched a shopkeeper's van full of goods in front of a shop.”

Participants thus had the chance to continue the preamble in at least two ways. One in which the ambiguous noun was interpreted as the patient (which would correspond to the misanalysis of the related garden‐path sentence) and another one, where it was interpreted as the beneficent (which would correspond to the correct analysis). We manually coded all the responses and calculated the proportion of continuations in which the ambiguous region was interpreted in line with the misanalysis (clozeambmis) and those in line with the correct analysis (clozeambcor).

The cloze scores in this case do not capture the likelihood or predictability of the upcoming content but rather the likelihood with which a certain analysis of the already read segment appears. In other words, it shows how often the ambiguous region was interpreted in a certain way. In this way, it differs from surprisal estimates, which are based on the probability of the upcoming word based on the preceding context (in this case, surprisal would show how likely the word “obchodnici” is to appear after “Luděk prohledal…”, or how likely the word “dodávku” is to appear after “Luděk prohledal obchodnici před prodejnou…”). This measure is also more general than, for example, structural bias or plausibility, since it reflects all the potential properties that may contribute to the decision leading the participants to ultimately choose one of the possible interpretations of the ambiguous region, not just the influence of one variable. However, the other predictors we examine (especially surprisal and plausibility) seem to play a role in this decision, as suggested by the moderate correlations with cloze scores (cf. the correlation matrix in 2).

**Fig. 2:**
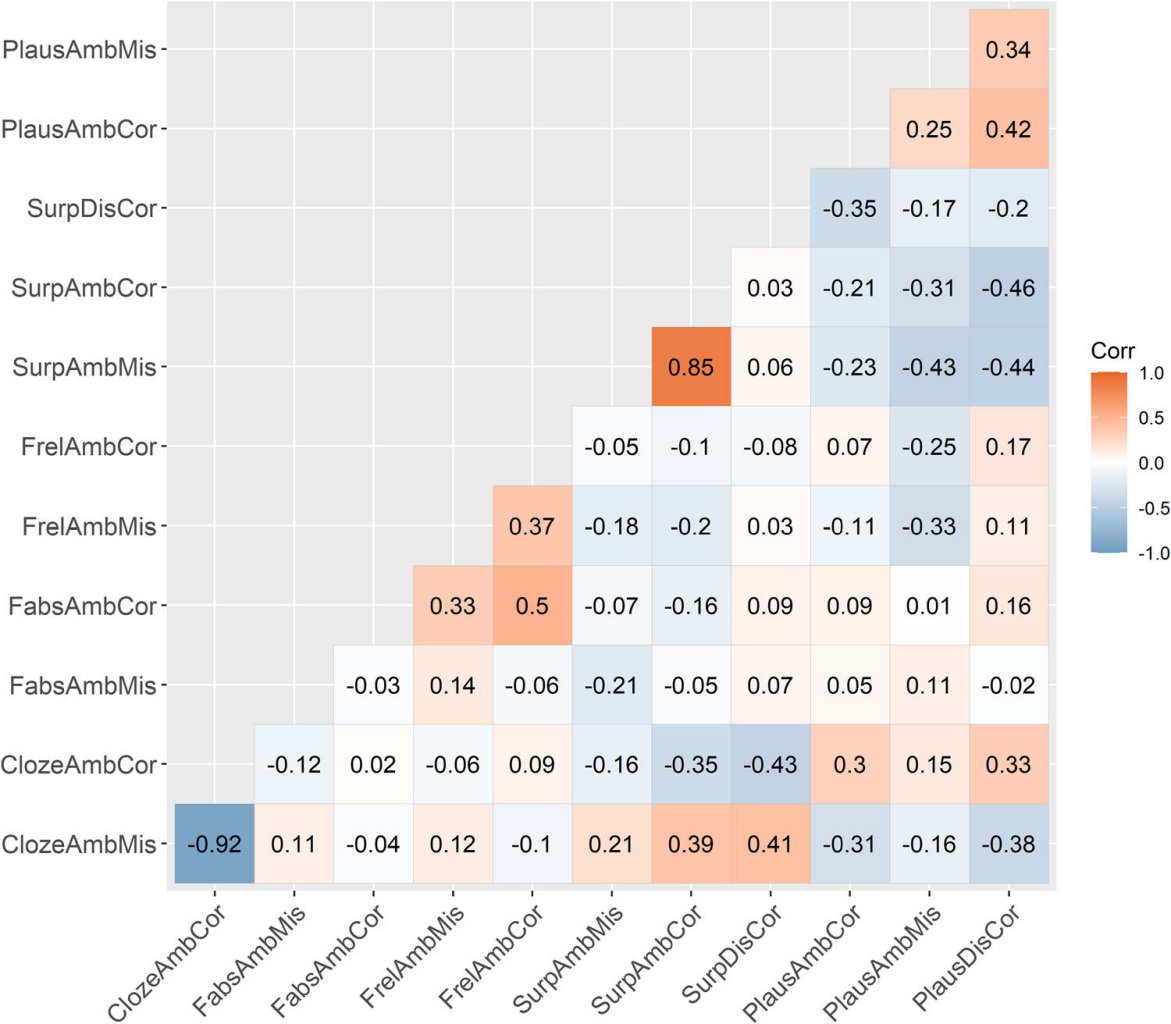
Pearson's correlation coefficients between the 12 variables of interest.

The only measure (apart from the corpus frequencies) that does not seem to correlate with the cloze scores at all is the plausibility of the misanalysis, which probably makes sense since our data lacked any clearly implausible misanalyses that could block the garden‐path effect from appearing in the first place (we wanted to make sure people get garden‐pathed). Other factors, such as verb bias or the need to shorten dependencies, may then play a bigger role than the plausibility of the scenario (especially when the situation does not describe an obvious nonsense), as was shown by, for example, Garnsey et al. (1997).

#### 2.3.2 Structural bias

To collect verb/structural bias data, we used the Czech version of the InterCorp v16ud corpus (Čermák & Rosen, 2012). We were interested in how often a given verb lemma occurs in combination with morphosyntactic structures consistent with either the initial misanalysis or the correct analysis of the ambiguous region.

For example, for sentence (16), we looked for occurrences of the lemma *prohledat* (“to search”) followed by a noun in the accusative case, which served as a direct object of the verb (misanalysis), and the same lemma followed by a noun in the dative case, which served as an adjunct (correct analysis). The results were manually checked, and unrelated occurrences were excluded.

We used two measures: absolute frequency (fabsambmis, fabsambcor), that is, the raw number of relevant occurrences in the whole corpus, and relative frequency (frelambmis, frelambcor), that is, the proportion of relevant occurrences relative to all occurrences of the given verb lemma.

#### 2.3.3 Surprisal

To calculate the surprisal estimates, we used the surprisal package in Python (Sathe, 2025) and a publicly available CzeGPT‐2 generative transformer model (Hájek & Horák, 2024), which has 124 million trainable parameters and was trained on 5 GB of Czech documents sourced from the internet.

Surprisal for individual words in each sentence was computed as the sum of the surprisal values of the corresponding subword tokens.

We specifically focused on three sentence regions. Following Huang et al. (2024), we calculated surprisal for the disambiguating region in the garden‐path sentences (surpdiscor). Additionally, analogous to the cloze task and frequency measures, we also computed surprisal values for the initial misanalysis of the ambiguous region (surpambmis), represented by the ambiguous noun phrase in the garden‐path condition (e.g., *Luděk prohledal obchodnici* ‐ “Luděk searched the shopkeeper [acc.sg.fem]”), and for the correct analysis of the ambiguous region (surpambcor), represented by the ambiguous noun in the non‐garden‐path condition (e.g., *Luděk prohledal obchodníkovi* ‐ “Luděk searched the shopkeeper [dat.sg.masc.]”).

Each region corresponded to a single word, which could consist of multiple subword tokens. The number of subword tokens varied across stimuli. The surprisal value for each word was calculated as the sum of the surprisals of its constituent subword tokens.

#### 2.3.4 Plausibility

We also collected plausibility ratings for the final list of sentences. The 66 experimental items were rated together with 60 fillers. The fillers consisted of 20 highly plausible sentences (e.g., “A well‐liked dean rewarded a student at a conference.”), 20 highly implausible sentences (e.g., “A young zebra hunted a lioness in a savanna.”), and 20 sentences describing unusual, but possible scenarios (e.g., “Mark's classmate is a junior world champion in curling.”). In total, participants rated 126 sentences altogether.

The rating task was administered online via the PCIbexFarm platform. A total of 111 Charles University students participated for course credit.

Similarly to the acceptability ratings, participants rated each sentence using a 100‐point slider scale. The scale endpoints were defined as “completely implausible” and “completely plausible.” A “completely plausible” sentence was described as one that seems sensible and likely in terms of its content, referring to a situation that could plausibly occur in the given context. A “completely implausible” sentence was described as one whose content is nonsensical or weird, referring to highly unlikely or impossible scenarios.

We employed a 3x2 design distributed across participants using a Latin square. Each participant rated (i) a potential misanalysis of the ambiguous region in both the garden‐path and non‐garden‐path condition (e.g., *Luděk prohledal před prodejnou obchodnici/obchodníka*: ʼʼLuděk searched a shopkeeper in front of a shop[acc.sg.fem./acc.sg.masc]), (ii) the correct interpretation of the disambiguating region in both the garden‐path and non‐garden‐path conditions (e.g., *Luděk prohledal před prodejnou dodávku se zbožím*: “Luděk searched a van full of goods in front of a shop”), and (iii) the full interpretation of the entire sentence in both conditions.

We were specifically interested in the misanalysis of the ambiguous region in the garden‐path condition (plausambmis), as this is the measure most commonly used in studies examining the effects of plausibility on garden‐path processing. Following the rationale outlined in Section 2.2, we also examined the plausibility of the correct analysis of the disambiguating region (plausdiscor) and the plausibility of the correct interpretation of the ambiguous region in the context of the full non‐garden‐path sentence (plausambcor).

#### 2.3.5 Relation between the predictors

The average values and other descriptive statistics for all predictors are reported in Table 1.

**Table 1:** Basic descriptive statistics for the variables of interest used in the analyses

Predictor	Mean	SD	Median	First quartile	Third quartile
clozeambmis	0.766	0.27	0.887	0.609	0.974
clozeambcor	0.268	0.27	0.139	0.043	0.487
fabsambmis	3702.333	8964.05	780.5	115	3085
fabsambcor	26.515	88.77	3	1	11
frelambmis	0.131	0.15	0.052	0.015	0.211
frelambcor	0.006	0.03	0	0	0.001
surpambmis	13.399	6.47	12.406	8.461	16.530
surpdiscor	11.972	3.01	12.162	10.023	13.672
surpambcor	16.563	6.02	15.573	13.048	18.646
plausambmis	70.109	22.64	75.03	52.88	90.94
plausdiscor	75.562	17.87	80.305	65.07	90.24
plausambcor	61.378	18.87	64.81	52.87	76.06

One of the problems for the analysis lies in the fact that there are many moderate and even strong correlations between these variables, as shown by Fig. 2. The problem of possible collinearity is addressed below in Section 3.7.

### 2.4 Hypotheses

For all the variables of interest, we examine their interaction with ambiguity–that is, we expect these variables to affect the garden‐path condition exclusively or more strongly than the non‐garden‐path condition. We are specifically interested in their effects on response accuracy to comprehension questions targeting the initial misanalysis, as well as on RTs in the disambiguating and/or spillover region.

We hypothesize that the more likely, frequent and plausible, or the less surprising the initial misanalysis is, the stronger the garden‐path effect will be. In such cases, reanalyzing the sentence correctly and inhibiting the initial misanalysis will be more difficult and time‐consuming. Conversely, the more likely, frequent and plausible and the less surprising the correct analysis of the ambiguous region and/or the correct analysis of the disambiguating region is, the easier and faster the reanalysis should be, leading to better comprehension outcomes.

Specifically, we expect higher values of the following predictors to be associated with longer RTs and lower response accuracy:

- Cloze probability of the initial misanalysis of the ambiguous region (clozeambmis)
- Absolute and relative frequency of the initial misanalysis of the ambiguous region (fabsambmis, frelambmis)
- Surprisal of the correct analysis of the ambiguous region (surpambcor)
- Surprisal of the disambiguating region (surpdiscor)
- Plausibility of the initial misanalysis (plausambmis)

By contrast, higher values of the following predictors are expected to result in shorter RTs and higher response accuracy:

- Cloze probability of the correct analysis of the ambiguous region (clozeambcor)
- Absolute and relative frequency of the correct analysis of the ambiguous region (fabsambcor, frelambcor)
- Surprisal of the initial misanalysis of the ambiguous region (surpambmis)
- Plausibility of the correct interpretation of the disambiguating region (plausdiscor)
- Plausibility of the correct interpretation of the ambiguous region (plausambcor)

## 3 Method

### 3.1 Data availability

All materials, analyses (including the R script), and preregistration can be found here: https://tinyurl.com/d9mu7hpe.

### 3.2 Ethics approval

Research ethics board approval for this study was acquired from the Research Ethics Committee, Faculty of Arts, Charles University (Ref. No.: UKFF/624176/2023). Participation in the experiments was voluntary, and all participants provided written informed consent. All data used in the current analyses were fully anonymized.

### 3.3 Participants

The initial sample included 303 native Czech speakers (undergraduate students at Charles University). Eight participants were excluded from the analysis—six due to low response accuracy on filler items (i.e., fewer than 75% correct responses), and two due to a high proportion of extremely short RTs (more than 5% of RTs below 100 ms), suggesting that they had clicked through a substantial portion of the experiment.

As a result, data from 295 participants were analyzed (244 female, 47 male, 3 nonbinary, and 1 undisclosed), with a mean age of 23.22 years. All participants received course credit for their participation.

### 3.4 Procedure

The experiment was conducted online using PC Ibex Farm (Zehr & Schwarz, 2018). After reading the initial instructions and providing informed consent, participants answered basic demographic questions (age, gender, native language, and presence of reading difficulties such as dyslexia).

The reading part of the experiment then began. We employed a moving‐window, word‐by‐word self‐paced reading task. Participants revealed each word in a sentence by pressing the space bar. Each keypress displayed the next word and simultaneously masked the previous one. RTs for each word were recorded. After reading each sentence, participants answered a yes‐no comprehension question by clicking with the mouse.

### 3.5 Materials

We worked with 66 Czech garden‐path sentences which are described in detail in the Supplementary Appendix. The sentences were matched in length and all had relatively short ambiguous regions (1–3 words). Each type was represented by six items, selected based on their naturalness ratings (see Section 2.1). Each item had an ambiguous (garden‐path) and unambiguous (non‐garden‐path) condition, which differed from each other only in the properties of the potentially ambiguous word. Participants only saw one condition of each item, based on a Latin‐square design. Each item was followed by a yes‐no comprehension question targeting the initial misanalysis of the ambiguous region. The correct response to this question was always “no.”

Overall, participants were presented with 66 experimental items and 78 fillers, all of which were grammatical, plausible sentences of varying lengths. Sentences were presented in random order. The total number of “yes” and “no” responses was balanced throughout the experiment.

Unfortunately, item092 had to be excluded from the analysis because the script mistakenly included a different question instead of the one targeting initial misanalysis.

### 3.6 Independent variables

The independent variables and the process of their collection are described in detail in Section 2.3. Based on the existing literature, we selected four potential predictors of the effect sizes on RTs and response accuracy: cloze task proportions, corpus frequencies, surprisal, and plausibility ratings. We focused on several parts of the sentence: the initial misanalysis of the ambiguous region, the correct analysis of the ambiguous region (often represented by the equivalent of the ambiguous region in the non‐garden‐path condition), and the correct analysis of the disambiguating region.

### 3.7 Data analysis

First, we checked participants' accuracy on filler items. Participants with a response accuracy below 75% were excluded from subsequent analyses (see Section 3.3).

Second, RT trimming was performed. RTs below 100 ms and above 5000 ms were excluded. In total, 0.38% of the RTs were excluded. The remaining RTs were log‐transformed.

Third, since we observed many correlations ranging from moderate to very strong among the predictors (see Fig. 2), we had to deal with the problem of predictor collinearity in modeling. We thus tested the effects of individual predictors in separate models and compared model fit using BIC (Schwarz, 1978). This allowed us to evaluate both whether each predictor influenced the dependent variable and which model provided the best fit.

For the analysis of response accuracy, we used logit mixed‐effects models (Jaeger, 2008). Each model included one predictor in interaction with ambiguity presence (i.e., whether the sentence was a garden‐path sentence). Ambiguity presence was sum contrast coded, with garden‐path sentences coded as 0.5 and non‐garden‐path sentences as –0.5. Random effects included both participant and item, with the random slope structure determined based on the recommendations by Matuschek (Matuschek, Kliegl, Vasishth, Baayen, & Bates, 2017).

RT analysis was conducted using linear mixed‐effects models with the lme4 package (Bates, Mächler, Bolker, & Walker, 2014). *p*‐values were estimated using Satterthwaite's approximations from the lmerTest package (Kuznetsova, Brockhoff, Christensen, & others, 2017). The fixed‐ and random‐effects structures were identical to those used in the response accuracy models. In the models, we focused on the disambiguating region and the immediately following region (spillover) where the typical garden‐path effect in processing would be expected.

Since we ran 12 parallel models for each variable, we had to account for inflating the risk of Type I error (see von der Malsburg & Angele, 2017). Therefore, we applied Bonferroni correction (Bonferroni, 1936) to each of the models. Thus, the *p*‐value significance threshold was reduced to 0.004 (i.e., 0.05/12) for the response accuracy analysis and to 0.002 (i.e., 0.05/24) for the RTs analysis. Only effects below this number are reported in the text (full models are available in Supplementary Materials).

## 4 Results

### 4.1 Response accuracy

**Fig. 3:**
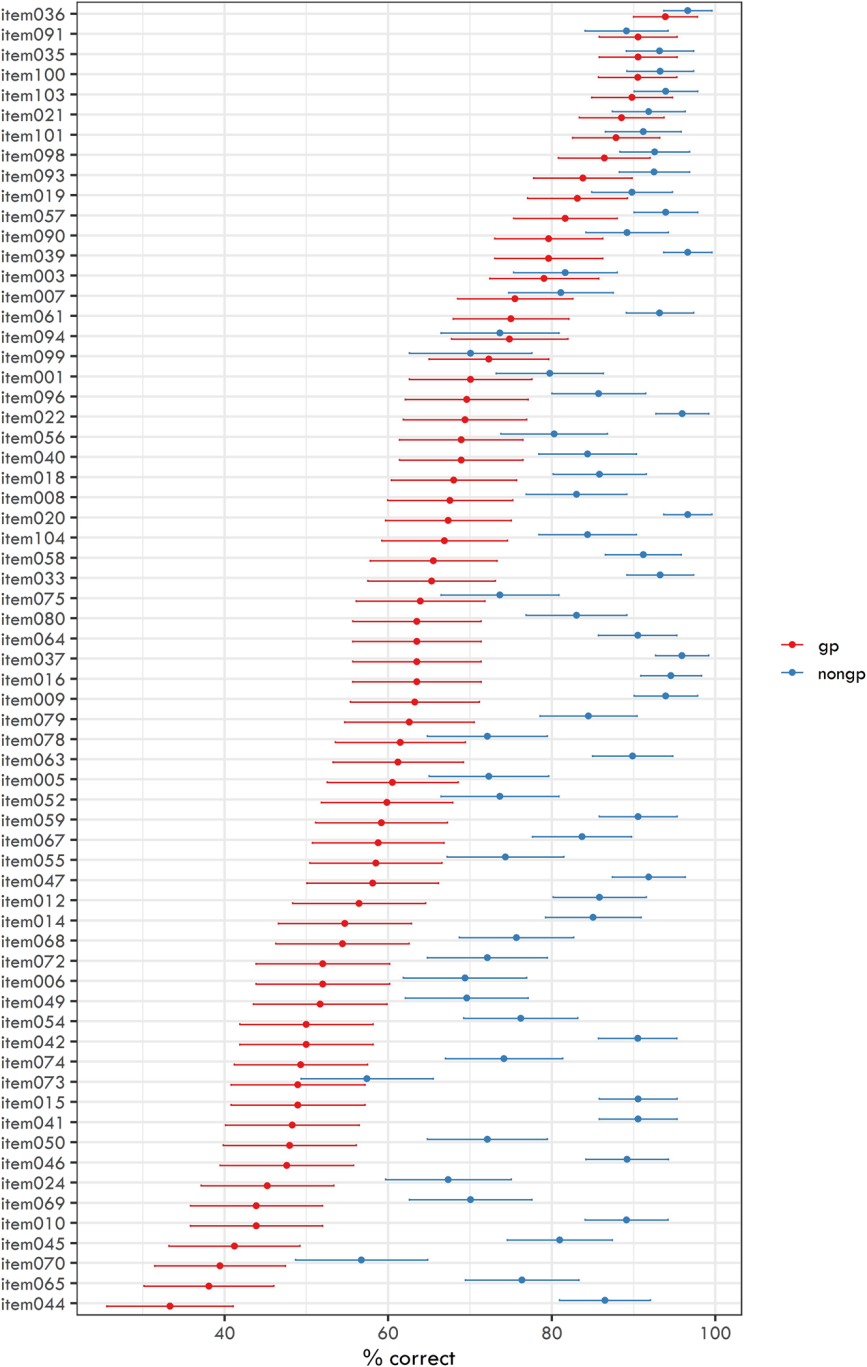
Response accuracy for individual items in two conditions (garden‐path and non‐garden‐path). Items are sorted in descending order based on their accuracy for garden‐path conditions.

The average response accuracy for individual experimental stimuli ranged from 33.33% to 96.62% with a mean score of 74.02% correct answers. Fig. 3 captures the response accuracy variability between items sorted in descending order based on the accuracy for garden‐path conditions.

As described in Section 3.7, we ran separate models for each independent variable in interaction with ambiguity presence (garden‐path vs. non‐garden‐path). Crucially, we were looking for significant interaction effects, which would show us a different effect of the predictor for garden‐path and non‐garden‐path sentences.

**Fig. 4:**
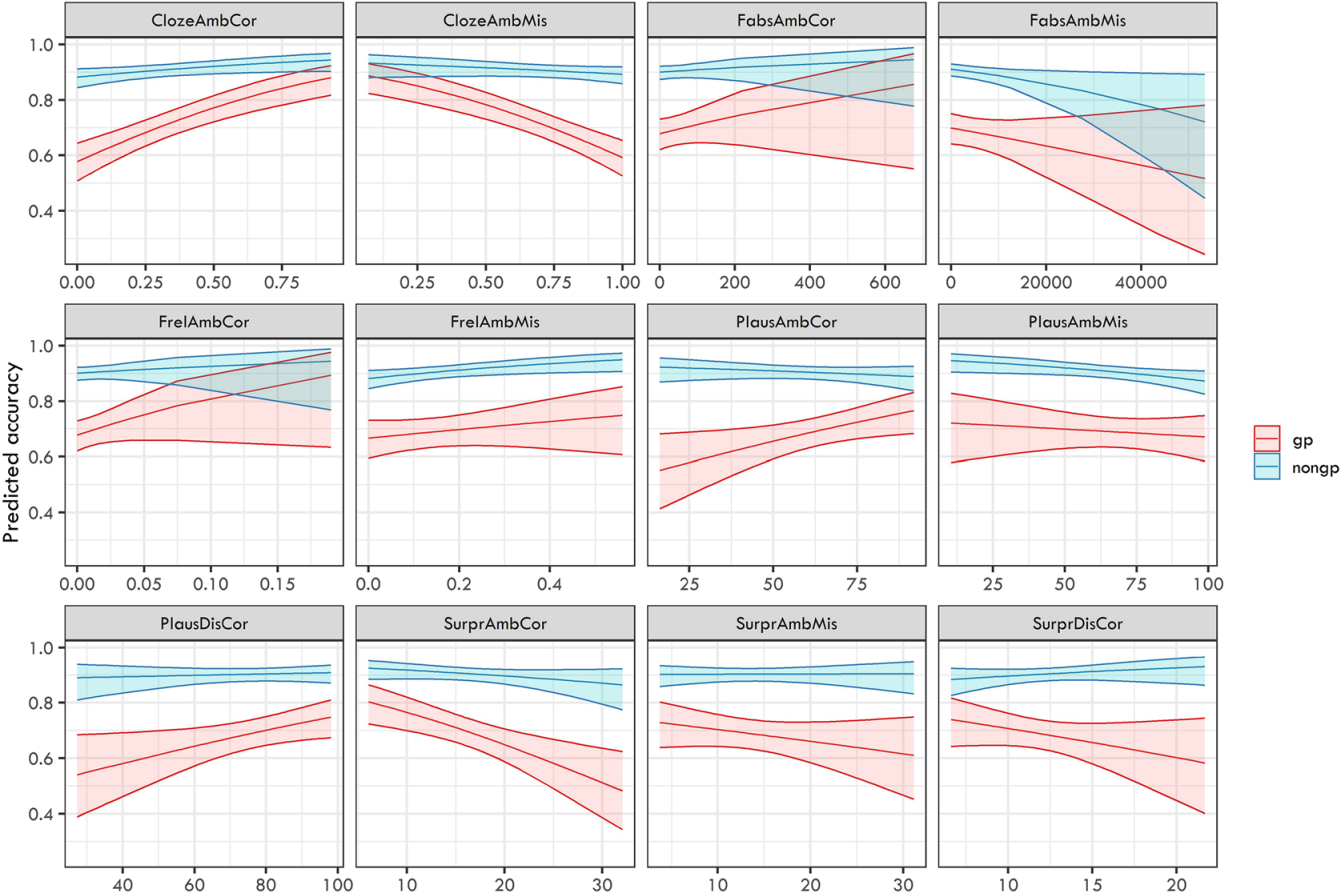
Plotted model estimates from the 12 logit mixed‐effects models targeting response accuracy as a dependent variable with individual predictors in interaction with ambiguity presence as fixed effects.

The estimates of the 12 logit mixed‐effects models are presented in Fig. 4. In all the models, we always documented a strongly significant effect of ambiguity presence (i.e., worse response accuracy for garden‐path structures than for non‐garden path controls).

However, we documented only two significant interaction effects after applying Bonferroni correction, namely, for models comprising (i) clozeambmis, that is, cloze probability for misanalysis (β=−0.344,SE=0.1,z=−3.446,p<.001), (ii) plausambcor, that is, plausibility of the correct analysis of the ambiguous region (β=0.342,SE=0.1,z=3.433,p<.001). None of the other models yielded significant interaction effects after applying the Bonferroni correction. Furthermore, significant main effects were yielded only in models with clozeambmis (β=−0.321,SE=0.1,z=−3.475,p<.001) and clozeambcor (β=0.366,SE=0.09,z=4.071,p<.001).

In sum, many models completely failed to produce desired effects, albeit small. In Table 2, we report model fit comparison using BIC (Schwarz, 1978). Models with both cloze probabilities are evaluated as the best ones, and based on the comparison, these two models fully account for the evidence among the models tested.

**Table 2:** Response accuracy models comparison using BIC

Predictor	LL	BIC	Delta BIC	BICWt
clozeambcor	−8788.36	17,704.93	0	0.58
clozeambmis	−8788.69	17,705.58	0.66	0.42
plausambcor	−8796.93	17,722.06	17.14	0
frelambmis	−8811.92	17,722.45	17.53	0
fabsambmis	−8812.22	17,723.04	18.12	0
frelambcor	−8813.11	17,724.83	19.91	0
fabsambcor	−8813.73	17,726.07	21.15	0
surpambcor	−8801.09	17,730.38	25.45	0
surpambmis	−8805.57	17,739.34	34.41	0
surpdiscor	−8807.51	17,743.21	38.29	0
plausambmis	−8809.6	17,747.39	42.47	0
plausdiscor	−8810.81	17,749.81	44.88	0

*Note*: Models are sorted based on the Delta BICc values.

### 4.2 Reaction times

**Fig. 5:**
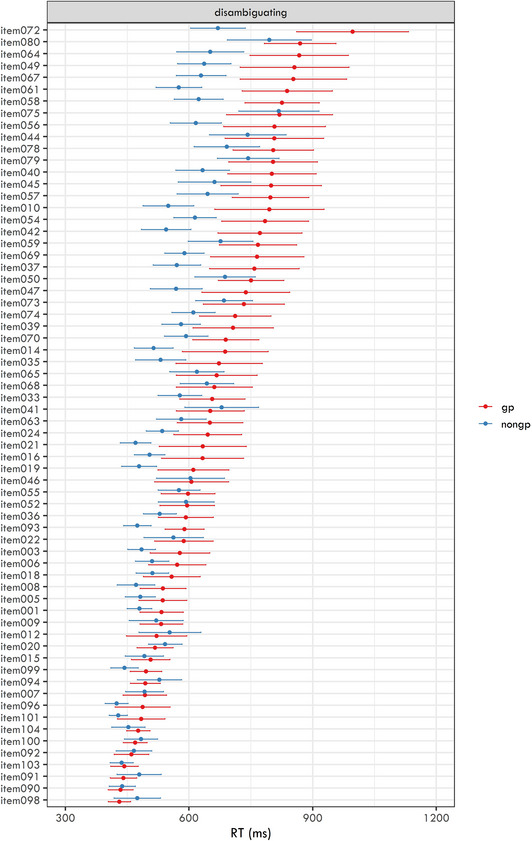
Mean reaction times for the disambiguating region in each item in the two conditions (garden‐path and non‐garden path). The error bars represent 95% confidence intervals of the means. Items are sorted in descending order based on their mean reaction time for garden‐path conditions.

The average RTs and their 95% confidence intervals for each item are plotted in Figs. 5 (disambiguating region) and 6 (spillover region).

**Fig. 6:**
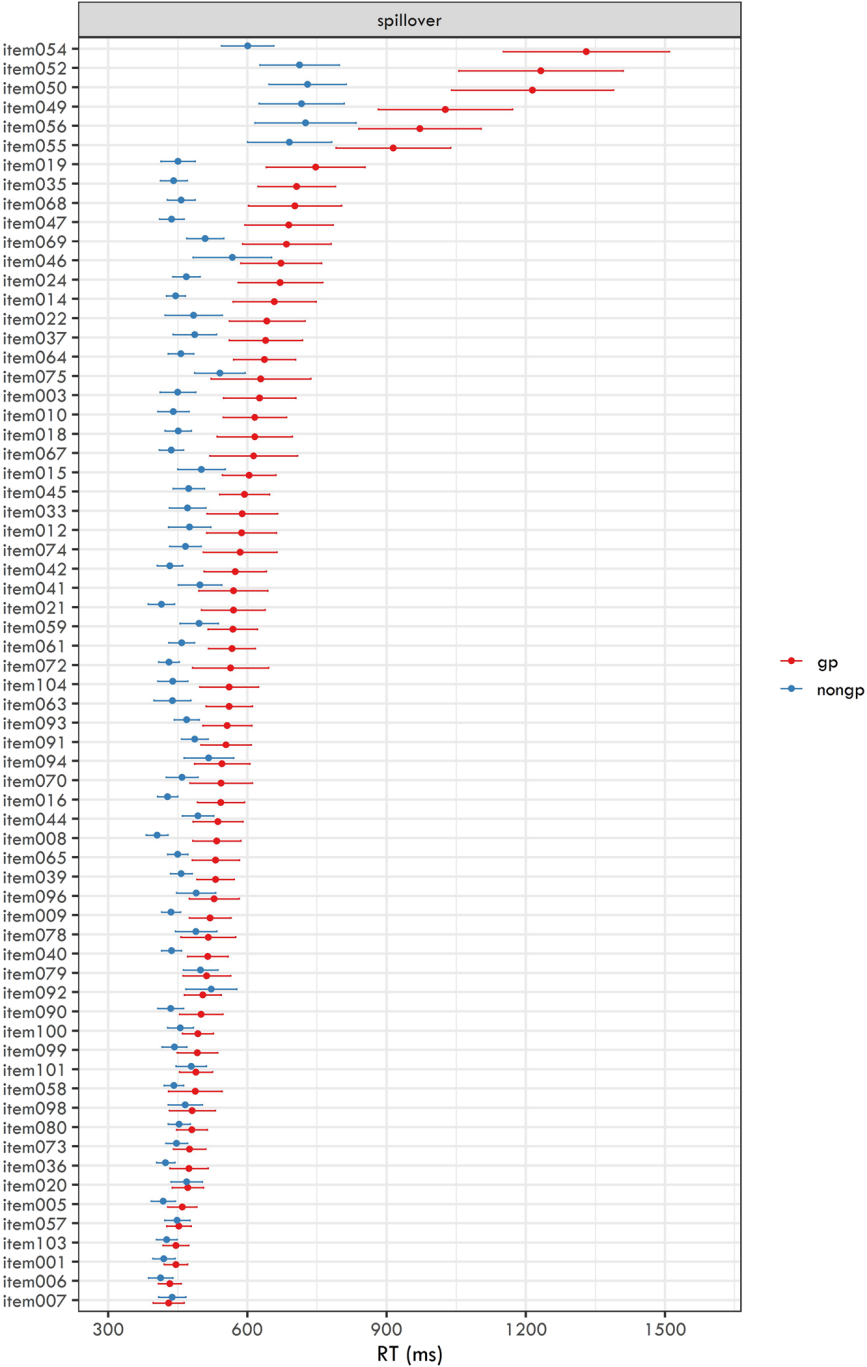
Mean reaction times for the spillover region in each item in the two conditions (garden‐path and non‐garden path). The error bars represent 95% confidence intervals of the means. Items are sorted in descending order based on their mean reaction time for garden‐path conditions.

**Fig. 7:**
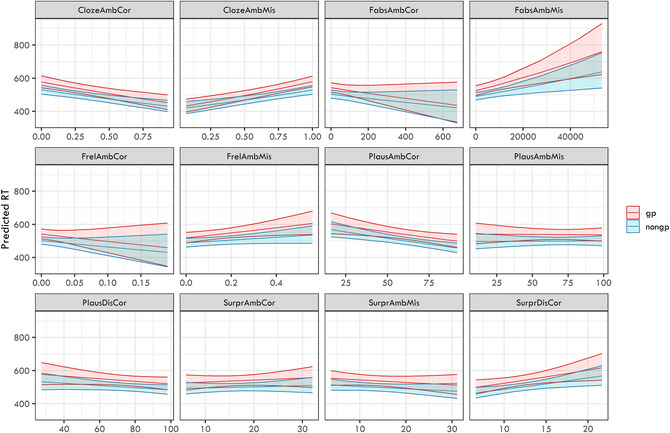
Plotted model estimates from the 12 linear mixed‐effects models targeting reaction times on the disambiguating region as a dependent variable with individual predictors in interaction with ambiguity presence as fixed effects.

The analysis was done separately for both regions: (i) disambiguating word and (ii) the spillover (i.e., the word following the disambiguating one). We again ran separate models for each independent variable in interaction with ambiguity presence. We were especially interested in the interaction effects, which would show the different role of the given predictor for garden‐path and non‐garden‐path structures. The estimates of the 12 linear mixed‐effects models are presented in Figs. 7 (disambiguating word) and 8 (spillover word). We documented a clear main effect of ambiguity on both the ambiguous and disambiguating regions in all models.

**Fig. 8:**
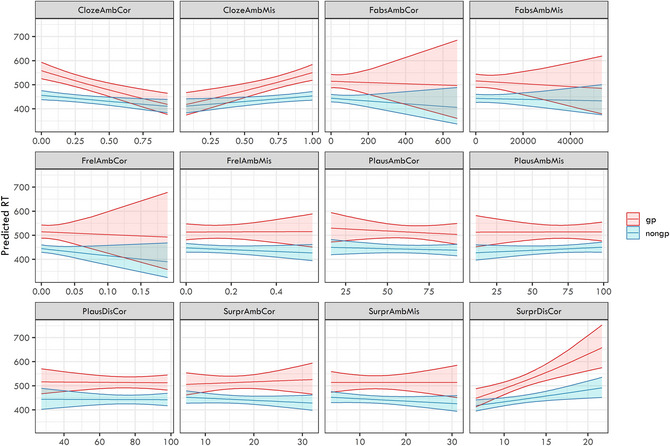
Plotted model estimates from the 12 linear mixed‐effects models targeting reaction times on the spillover region as a dependent variable with individual predictors in interaction with ambiguity presence as fixed effects.

We found a significant interaction of clozeambmis with ambiguity for the disambiguating region (β=0.018,SE=0.006,t=3.320,p<.001). Moreover, this region yielded three significant main effects, namely, of (i) clozeambmis (β=0.075,SE=0.015,t=5.206,p<.001), (ii) clozeambcor (β=−0.066,SE=0.015,t=−4.315,p<.001), (iii) fabsambmis (β=0.053,SE=0.016,t=3.344,p=.001). The model comparisons are presented in Table 3. The models using clozeambmis, plausambcor, clozeambcor, and fabsambmis received the highest support and were thus evaluated as the best ones given the data.

**Table 3:** Comparison of the linear mixed effects models for reaction times on the disambiguating region using BIC

Predictor	LL	BIC	Delta BIC	BICWt
clozeambmis	−9633.90	19,356.65	0	0.41
plausambcor	−9624.23	19,357.05	0.40	0.34
clozeambcor	−9624.58	19,357.75	1.10	0.24
fabsambmis	−9627.20	19,363.00	6.35	0.02
surpdiscor	−9629.51	19,367.62	10.96	0
plausdiscor	−9631.77	19,372.13	15.48	0
frelambcor	−9632.07	19,372.74	16.08	0
surpambmis	−9632.18	19,372.96	16.31	0
surpambcor	−9632.63	19,373.86	17.21	0
plausambmis	−9632.74	19,374.07	17.42	0
frelambmis	−9717.77	19,524.39	167.74	0
fabsambcor	−9719.67	19,528.20	171.54	0

*Note*: Models are sorted based on the Delta BICc values.

Importantly, three models of the spillover region yielded significant interaction effects after applying Bonferroni correction, namely, those using cloze scores and surprisal of the disambiguating region as predictors. In the case of the cloze probability of the misanalysis (clozeambmis), the interaction was positive (β=0.051,SE=0.012,t=4.384,p<.001), meaning a larger positive slope for garden‐path sentences (i.e., the more the misanalysis was probable, the slower the reactions were on the word following the disambiguating word). The opposite tendency was found for clozeambcor, that is, cloze probability of the correct analysis, where the interaction was negative (β=−0.053,SE=0.012,t=−4.593,p<.001)—the more this analysis was probable, the faster the garden‐path sentences were processed on the spillover word. For surpdiscor, surprisal of the disambiguating region, higher surprisal values lead to higher RTs (β=0.044,SE=0.012,t=3.642,p<.001).

Three significant main effects were also documented, namely, of clozeambmis (β=0.054,SE=0.015,t=3.508,p<.001), clozeambcor (β=−0.058,SE=0.015,t=−3.795,p<.001), and surpdiscor (β=0.055,SE=0.015,t=3.542,p<.001). Other predictors failed to reach significance after applying the Bonferroni correction.

The comparison of the models of the spillover region using BIC is presented in Table 4. We can see two models being supported by the comparison (the models using cloze scores). The model with clozeambcor score has the best weight (0.71), followed by the model with clozeambmis (0.29).

**Table 4:** Comparison of the linear mixed effects models for reaction times on the spillover region (i.e., the word directly following the disambiguating one) using BIC

Predictor	LL	BIC	Delta BIC	BICWt
clozeambcor	−8218.34	16,545.28	0	0.71
clozeambmis	−8219.22	16,547.04	1.76	0.29
frelambcor	−8225.61	16,559.82	14.53	0
surpambmis	−8225.73	16,560.07	14.79	0
frelambmis	−8226.02	16,560.65	15.37	0
plausambmis	−8226.15	16,560.90	15.62	0
fabsambcor	−8226.92	16,562.45	17.17	0
surpdiscor	−8315.69	16,720.24	174.96	0
surpambcor	−8319.50	16,727.87	182.59	0
plausambcor	−8322.49	16,733.85	188.56	0
fabsambmis	−8322.53	16,733.91	188.63	0
plausdiscor	−8375.30	16,839.46	294.18	0

*Note*: Models are sorted based on the Delta BICc values.

## 5 Discussion

Our study examined the effects of cloze probability, structural/verb bias, surprisal, and plausibility on garden‐path effects, as measured by reading times and response accuracy. These factors have previously been proposed as relevant to resolving local syntactic ambiguities, though prior studies have yielded mixed results.

By analyzing a diverse set of locally ambiguous and unambiguous sentences, we found robust and consistent effects of cloze probability. These effects were documented in both response accuracy and reading times, including significant main effects and interactions. Models that included cloze scores consistently showed superior fit based on BIC. In contrast, structural bias, surprisal, and plausibility showed weaker, inconsistent or nonsignificant effects.

For response accuracy, we observed a key interaction between ambiguity and clozeambmis, indicating that the more likely the misanalysis was to occur, the more likely participants were to arrive at an incorrect interpretation, which persisted even after potential reanalysis. This suggests that strongly preferred continuations are more difficult to override once a disambiguating cue is encountered.

Another significant interaction involved plausambcor: when the intended interpretation of the ambiguous region was highly plausible, participants were more successful in resolving the ambiguity and avoiding/inhibiting the misanalysis.

We interpret these results as evidence that garden‐path disambiguation is shaped by a combination of several factors, taking into account syntax, semantics, and pragmatics, rather than by general frequency‐based structural preferences or semantics/pragmatics in isolation. While structural/verb bias, as indexed by frequency, has been argued to guide initial parsing decisions, our data suggest that the key factor influencing reanalysis is in fact the general likelihood with which the misanalysis occurs in the first place, which is shaped by all properties of the preceding context (and not its structural, lexical, or pragmatic bias in isolation).

This interpretation is supported by the RT data. Models including cloze scores showed significant interactions with ambiguity presence, indicating a different slope for locally ambiguous sentences: the more the cloze score favored the misinterpretation, the longer the RTs were.

Importantly, the only other significant interaction effects we observed in terms of RTs is the effect of surprisal (namely, surpdiscor) on the spillover region. The remaining predictors—surprisal of the ambiguous region, structural bias, and plausibility—failed to yield the expected effects.

A natural question raised by our results is why cloze scores perform better than surprisal in our experiments, given that both measures are intended to quantify predictability. Conceptually, cloze scores and surprisal both integrate multiple sources of linguistic information (syntactic, semantic, pragmatic, and distributional) that comprehenders may rely on when committing to an interpretation. The moderate correlations between the two measures in our data (see Fig. 2) further illustrate that they tap into related underlying process.

A most likely explanation for the difference in effects relates to how these predictors are operationalized. Surprisal estimates quantify how likely a particular word is to occur next, given the preceding context. In contrast, the cloze probabilites used in our study quantify how likely participants are to choose a particular interpretation of an entire sentence fragment (e.g., *While Anna dressed the baby…*) *after reading it*. In this sense, cloze scores reflect the distribution of comprehenders' preferred interpretations, whereas surprisal reflects the model‐derived probability of specific lexical continuations (e.g., *dressed*).

Other differences relate to how surprisal and cloze scores were collected/computed. For example, cloze scores are subject to task‐related constraints (e.g., limited sample size, omission of low‐probability continuations) that can distort the underlying probability distribution (Smith & Levy, 2011; Shain, Meister, Pimentel, Cotterell, & Levy, 2024). Large‐language‐model‐based surprisal, by contrast, assigns nonzero probability to any lexical continuation but reflects the statistical properties of the model's training corpus rather than human expectations per se. In our case, cloze scores indexed the preferred structural interpretation rather than lexical predictability, and were, therefore, not affected by the zero‐probability issue typical of completion tasks.

Nonetheless, our cloze data may reflect other task‐related influences. Because cloze score is an offline measure, participants' completions may be shaped by strategic or post‐hoc reasoning (de Varda, Marelli, & Amenta, 2024). For instance, participants might initially favor an ambiguous interpretation but then settle on a disambiguated one that is easier to continue (Ceháková & Chromý, 2023). A similar process may occur in self‐paced reading, where the visible continuation can bias interpretation in comparable ways (de Varda et al., 2024).

It is also possible that the probability distributions comprehenders rely on are different from those generated by LLMs (de Varda et al., 2024; Smith & Levy, 2011; Shain et al., 2024), either due to various biases in the texts the LLMs are trained on, which might not properly reflect the actual language statistics “in the world,” or due to errors or individual biases in learning or processing of human participants, which might not be in line with the LLM‐generated distributions. The latter option is further supported by the fact that the fit of surprisal estimates to human data improves when memory constraints (Futrell, Gibson, & Levy, 2020), expertise (Škrjanec, Broy, & Demberg, 2023), or different emphasis on lexicon and syntax (Arehalli, Dillon, & Linzen, 2022) are taken into account.

Several studies (e.g., Huang et al., 2024; van Schijndel & Linzen, 2021) suggest that although prediction contributes to garden‐path effects, additional mechanisms are likely involved. Moreover, human predictions may differ from those generated by LLMs. For example, Arehalli et al. (2022) show that models produce RT predictions more closely aligned with human data when lexical and syntactic predictability are modeled independently and when syntactic information is weighted more strongly—although even in such cases, the size of the slowdown remains underestimated. Huang et al. (2024) further propose that ambiguity resolution involves reanalysis in addition to prediction, with extra processing costs associated with these processes. Another possibility is that the representations supporting prediction are disrupted by noise or memory limitations, as formalized in lossy‐context surprisal (Futrell et al., 2020). The potential role of memory is also supported by findings that longer ambiguous regions tend to be associated with larger garden‐path effects (Ferreira & Henderson, 1991; Christianson et al., 2001). Since our cloze scores are derived from human judgments, they are more likely to reflect the influence of these additional processes.

The finding that cloze scores are a stronger and more consistent predictor of garden‐path effects than surprisal estimates may appear related to previous work showing that surprisal underestimates the magnitude of such effects (e.g., Huang et al., 2024; van Schijndel & Linzen, 2021). However, the results of these studies are not directly comparable due to differences in methodologies: unlike these studies, we did not estimate the predicted effect size of the garden‐path effect. Our findings show only that surprisal contributes to the speed of disambiguation (but not to response accuracy). The magnitude of this contribution remains an open question.

With respect to plausibility, we observed a significant interaction between ambiguity and plausambcor in the accuracy data. This suggests that the semantic fit of the intended interpretation can facilitate recovery from misanalysis, potentially by reinforcing the correct structure once it becomes available. However, plausibility effects were generally weaker than those of cloze scores, and we found no significant effects of plausibility in the RT data. This pattern is consistent with findings from Qian et al. (2018) and Huang et al. (2024). Moreover, the absence of plausibility effects across a diverse set of sentence types aligns with results from Roberts & Felser (2011) and Nakamura & Arai (2016), who found plausibility effects only for certain sentence types—typically the more challenging ones.

One limitation of the present study concerns the measurement of the predictors. While our operationalizations may not fully capture all aspects of verb bias, cloze probability, plausibility, or surprisal, we believe the measures used are grounded in well‐established methodologies. Cloze scores and plausibility ratings were derived from responses by more than 100 native Czech speakers with a similar demographic profile to participants in the self‐paced reading task (i.e., undergraduate students at Charles University). The other two predictors (structural bias and surprisal) were computed using widely available tools: the InterCorp v16ud corpus (Čermák & Rosen, 2012) and the CzeGPT‐2 generative transformer model (Hájek & Horák, 2024), respectively. It is, however, possible that the genres represented in InterCorp or the training data for CzeGPT‐2 do not fully reflect the distributional properties of Czech as used in our experimental materials. If so, this may have introduced noise or confounds into the analyses, reducing our ability to detect relevant effects.

A further possible reason for the absence of, specifically, verb bias effects is that some frequency values (especially for the ambcor condition) were extremely low or even zero. This may have resulted in floor effects that complicated model estimation. Moreover, our frequency measures targeted morphosyntactic biases in isolation, without considering lexical context, whereas the cloze task, which showed the most robust effects, reflected prior context as well as lexical content of the ambiguous word. Based on our results, verb bias alone does not appear sufficient to explain the observed differences across items when using a structurally diverse stimulus set.

Another potential limitation concerns the scope and composition of our stimulus set. It is possible that some of the reported effects generalize only to the chosen subset of garden‐path structures, or that certain findings are specific to Czech. While the former cannot be ruled out, our stimulus set was explicitly designed to sample broadly from the space of locally ambiguous constructions. We included 11 types of garden‐path sentences that varied in syntactic structure, semantic interpretation, and naturalness, while controlling for factors such as sentence length and the length of the ambiguous region. The items also varied in their effect size, producing a wide range of processing difficulty across constructions. Importantly, we consistently observed main effects of ambiguity across models: participants showed increased reading times in the disambiguating and spillover regions in the garden‐path condition, and their responses to comprehension questions indicated lingering effects of initial misanalysis. These patterns confirm that the stimuli reliably elicited garden‐path effects, supporting the generalizability of our findings across a structurally diverse set of constructions.

The results may, however, be specific to the Czech language and Czech speakers. As mentioned earlier, Czech is a morphologically rich language with abundant case syncretism and relatively flexible word‐order. Both of these aspects may frequently create ambiguities, and, subsequently, impact how these ambiguities are treated by speakers. For example, Czech speakers might be prone toward relying on all potential sources of information in order to disambiguate structures rather than relying on structural biases or plausibility information in isolation (since these might often prove inefficient by themselves due to the numerous potential sources of ambiguity in Czech). Consequently, some of the factors which did not turn out to be successful predictors of the garden‐path effects in Czech might in fact work well for other languages with more fixed word‐order (such as English) or less inflection (such as Mandarin). Such cross‐linguistic differences may offer another explanation for why, for example, verb‐bias turned out to be a good predictor of garden‐path effects in many studies working with English stimuli, but did not exert any effects in our experiment. Studying how various linguistic properties influence ambiguity resolution in different languages and showing what types of information speakers of these languages rely on could thus offer valuable insights and put the results of this paper in a wider perspective.

It is also worth emphasizing that our study extends prior work in how the relevant predictors are operationalized. In addition to examining properties of the initially preferred (misanalyzed) interpretation of the ambiguous region—an approach common in earlier studies—we also considered features of the correct interpretation, as well as properties of the disambiguating region itself. This approach reflects the view that both the strength of the initial misanalysis and the ease of identifying and creating the correct analysis jointly shape processing difficulty and reanalysis success in garden‐path sentences. Focusing on the properties of these regions is crucial, since both of them play an important role during reanalysis—the disambiguating region signals the error in the current analysis and sets the reanalysis in motion (cf. Fodor & Inoue, 2000, 1994), and the correct interpretation of the ambiguous region is what needs to be retrieved if the reanalysis is to succeed. It has repeatedly been shown that they can offer crucial insights in how reanalysis in garden‐path sentences work (e.g., some studies show that these regions often do not get analyzed correctly at all, despite researchers assuming the opposite) (Chromý, 2022; Ceháková & Chromý, 2025). Our study adds another piece of evidence of their relevance.

Finally, we wish to emphasize that the absence of significant effects on RTs or response accuracy does not imply that the predictors in question have no influence on the relative difficulty of garden‐path sentences. A growing body of research suggests that comprehension processes are highly individualized, and that behavioral measures such as processing speed or eye movements do not always provide a complete picture of the cognitive mechanisms involved in sentence interpretation. For instance, Christianson et al. (2017) argued that RTs in the disambiguating region do not reliably reflect whether reanalysis has been successful. Similarly, Paape and Vasishth (2022) and Christianson et al. (2024) demonstrated that individual comprehenders engage in distinct rereading strategies, which can lead to divergent comprehension outcomes. Moreover, Ceháková & Chromý (2023) showed that a single garden‐path structure can give rise to a wide variety of mental representations across participants.

In light of this variability, it is plausible that some predictors examined in our study may exert different effects on different individuals, or that these effects are obscured by interindividual differences in processing strategies. For example, a garden‐path sentence containing a highly plausible misanalysis may be difficult for some comprehenders but not for others. Among those who find it challenging, responses may vary widely: some may rapidly and successfully reanalyze the sentence; others may engage in effortful and prolonged revision; some may recognize the ambiguity but give up on resolving it; and still others may persist in misanalysis despite extended processing time. These qualitative differences in response profiles are likely to reduce the sensitivity of aggregate measures such as mean RT or accuracy, and may explain the apparent absence of effects for certain predictors.

Lastly, as some of the previous studies have shown (e.g., Garnsey et al., 1997; Nakamura & Arai, 2016; Roberts & Felser, 2011), some predictors may elicit significant effects only under specific conditions (for instance, within specific syntactic structures or in especially difficult sentences). It is also plausible that predictors interact: when comprehenders cannot rely on a strong structural bias to disambiguate a garden‐path sentence, they may instead rely more heavily on plausibility cues, or vice versa.

Unfortunately, a full investigation of cross‐construction differences or interactions among predictors lies beyond the scope of the present study. However, these questions are important ones, and they point toward several promising directions for future research.

## 6 Conclusions

Taken together, our results show that the general likelihood of misanalysis/correct analysis of the ambiguous region, as measured by cloze scores, is a reliable and consistent predictor of processing difficulty in garden‐path sentences, across both RTs and comprehension accuracy. These findings suggest that the general likelihood with which the misanalysis occurs, as shaped by a complex combination of linguistic properties (syntactic, semantic, pragmatic, contextual…)—rather than any of these properties in isolation—plays a dominant role in syntactic disambiguation. By systematically comparing multiple predictors across a range of constructions, our study also highlights the importance of using a diverse but well‐controlled stimuli sets in psycholinguistic research, as different types of sentences may vary in their outcomes.

## Funding

This work was supported by the European Regional Development Fund project “Beyond Security: Role of Conflict in Resilience‐Building” (reg. no.: CZ.02.01.01/00/22_008/0004595). Both authors were also supported by the Charles University institutional program Cooperatio.

## Supporting information

 
[Supplementary-material cogs70208-supl-0001]

