# Supplementary material for: Cloze, Frequency, Surprisal, or Plausibility? A Comparative Analysis of Predictors for Local Ambiguity Resolution
Source: Cogn Sci. 2026 Apr 30;50:e70208. doi: 10.1111/cogs.70208 (PMC13129637; doi:10.1111/cogs.70208)
Supplement: Supplementary file 1 [file COGS-50-e70208-s001.pdf]

Appendix A

Types of garden-path sentences

We worked with 11 types of Czech garden-path sentences, which were all carefully constructed to be relatively short, matched for length (8 words) and, as much as possible, for the length of the ambiguous region (the ambiguous and disambiguating region were separated by 1-3 words). Each sentence had a closely related non-ambiguous counterpart, which differed only in the gender or number of the ambiguous noun (occasionally a close synonym was used instead) or in the conjunction used in the stimulus.

The types of sentences we employed in the experiment, together with a brief description, glosses, translations, related comprehension questions and examples of the DisCor, AmbMis and AmbCor strings, are listed below.

Type I: wordorderobject

In this type, two nouns compete for the role of the subject/agent. The ambiguous noun standing at the beginning of the sentence is initially interpreted as its subject (nom.sg), since SVO is the more common word order in Czech, but can also be interpreted as an object (acc.sg.). The sentence is disambiguated when comprehenders encounter another noun in the nominative case, which can only be identified as the subject of the sentence. The ambiguous noun then needs to be reanalyzed as the object (acc.sg), e.g. *a restaurant welcomed something* vs. *a dad welcomed a restaurant*). In this type, the two competing nouns are not semantically similar – the ambiguous noun refers to an object/institution (e.g. the restaurant), while the disambiguating noun refers to a person (e.g. the dad). In the unambiguous condition, the ambiguous noun was replaced by a semantically similar noun which could never be identified as the subject of the verb.

(17) Restaurant-0/Restaurac-i nadšeně
Restaurant-NOM/ACC.M.SG/Restaurant-ACC.F.SG excitedly
uvíta-l i tatínek-0 se dvěma dětmi.
welcome-3SG.M.PST even dad-NOM.M.SG with two kids.
'Even the dad with two kids excitedly welcomed the restaurant.'

Question: Bylo něco uvítáno restaurací? (*Was something welcomed by the restaurant?*)

AmbMis: Restaurant něco nadšeně uvítal. (*The restaurant excitedly welcomed something.*)

DisCor: Tatínek se dvěma dětmi něco nadšeně uvítal. (*The dad with two kids excitedly welcomed something.*)

AmbCor: Restauraci nadšeně uvítal i tatínek se dvěma dětmi. (*Even the dad with two kids excitedly welcomed the restaurant.*)

Type II: wordorderperson

This type was syntactically identical to Type I, but it differed semantically. Here, the two competing nouns both referred to a person (and thus were semantically more similar).

(18) Prodejc-e/Prodejkyn-i	opakovaně	podved-l	i
Retailer-NOM/ACC.M.SG/retailer-ACC.F.SG	repeatedly	deceive-3SG.M.PST	even

dodavatel-0	při	předání	zboží.
supplier-NOM.M.SG	while	delivering	goods.

‘Even the supplier repeatedly deceived the retailer while delivering the goods.’

Question: Podvedl někoho prodejce? (*Did the retailer deceive someone?*)

AmbMis: Prodejce někoho opakovaně podvedl. (*The retailer repeatedly deceived someone.*)

DisCor: Dodavatel při předání zboží někoho opakovaně podvedl. (*The supplier repeatedly deceived someone while delivering the goods.*)

AmbCor: Prodejce opakovaně podvedl i dodavatel při předání zboží. (*Even the supplier repeatedly deceived the retailer while delivering the goods.*)

Type III: patinstr

In this type, two nouns compete for the role of the patient/theme. The ambiguous noun is homonymous in its ACC.PL. and inst.pl. form. It is initially interpreted as a patient (ACC.PL.), but needs to be reanalyzed as an instrument INST.PL.. The sentence is disambiguated when another noun appears, which can only be interpreted as the patient.

(19) Richard zakryl plakát-y/plakát-em s
Richard covered_up poster-ACC./INST.M.PL./poster-INST.M.SG with
celebritou nápis-0 na zdi.
celebrity writing-ACC.M.SG on wall.
'Richard covered up the writing on the wall with celebrity posters.'

Question: Zakryl Richard plakáty? (*Did Richard cover up the posters?*)

AmbMis: Richard zakryl plakáty s celebritou. (*Richard covered up the celebrity posters.*)

DisCor: Richard zakryl nápis na zdi. (*Richard covered up the writing on the wall.*)

AmbCor: Richard zakryl plakátem s celebritou nápis na zdi. (*Richard covered up the writing on the wall with a celebrity poster.*)

Type IV: patbenperson

Once again, two nouns are competing for the role of patient again. This time, the ambiguous noun is homonymous in its ACC.SG. and DAT.SG. form. It is initially analyzed as a patient/theme (ACC.SG.) but later needs to be reanalyzed as a beneficient/external possessor (DAT.SG.), when a noun that can only be identified as the actual patient is encountered. In this case, the ambiguous noun e always refers to a person, while the disambiguating noun refers to an inanimate object.

(20) Kamil polil vévodkyn-i/vévod-ovi na večírku
Kamil spilled duchess-ACC./DAT.F.SG./duke-DAT.M.SG at party
pohovk-u ze semiše.
sofa-ACC.M.SG from chamois.
'Kamil spilled [his drink] on the duchess'/duke's chamois sofa at the party.'

Question: Polil Kamil vévodkyni? (*Did Kamil spill his drink on the duchess?*)

AmbMis: Kamil polil na večírku vévodkyni. (*Kamil spilled his drink on the duchess at the party.*)

DisCor: Kamil polil na večírku pohovku ze semiše. (*Kamil spilled his drink on the chamois sofa at the party.*)

AmbCor: Kamil polil vévodovi na večírku pohovku ze semiše. (*Kamil spilled his drink on the duke's chamois sofa at the party.*)

Type V: patbenperson2

This type is syntactically identical to Type IV, but the two competing nouns both refer to a person and are thus semantically more similar.

(21) Samuel postřelil zrádkyn-i/zrádc-i ve vlaku
Samuel shot traitress-ACC./DAT.F.SG./traitor-DAT.M.SG on train
kamarádk-u z dětství.
friend-ACC.M.SG from childhood.
'Samuel shot the traitress'/traitor's childhood friend on a train.'

Question: Postřelil Samuel zrádkyni? (*Did Samuel shoot the traitress?*)

AmbMis: Samuel postřelil ve vlaku zrádkyni. (*Samuel shot the traitress on a train.*)

DisCor: Samuel postřelil ve vlaku kamarádku z dětství. (*Samuel shot a childhood friend on a train.*)

AmbCor: Samuel postřelil zrádci ve vlaku kamarádku z dětství. (*Samuel shot the traitor's childhood friend on a train.*)

Type VI: deverbaddr

In this type, there are two nouns competing for the role of an recipient. The ambiguous noun is homonymous in its DAT.SG. and INST.PL. form. It is initially interpreted as an adjunct of a verb, with the role of a recipient (DAT.SG.). However, when another noun appears which can only be identified as the recipient, the ambiguous noun has to be reanalyzed. In the intended analysis (INST.PL.) the ambiguous noun is in fact an argument (agent) of a verbal adjective which modifies the verb's object.

(22) Eliška radostně nabídla důchodc-i/důchodc-em
Eliška happily offered retiree-DAT.M.SG./INST.M.PL/retiree-INST.M.SG
upečené sušenky návštěvník-ovi bistra.
baked cookies visitor-DAT.M.SG bistro.
'Eliška happily offered the cookies baked by the retirees/retiree to the visitor of the bistro.'

Question: Nabídla Eliška sušenky důchodci? (*Did Eliška offer the cookies to the retiree?*)

AmbMis: Eliška radostně nabídla upečené sušenky důchodci. (*Eliška happily offered the baked cookies to the retiree.*)

DisCor: Eliška radostně nabídla upečené sušenky návštěvníkovi bistra. (*Eliška happily offered the baked cookies to the visitor of the bistro.*)

AmbCor: Eliška radostně nabídla důchodci upečené sušenky návštěvníkovi bistra. (*Eliška happily offered the cookies baked by the retiree to the visitor of the bistro.*)

Type VII: deverbpat

This type is similar to Type VI in the fact that the ambigous noun can once again be interpreted as an argument of a verb or of a verbal adjective. This time, however, the ambiguous and the disambiguating noun compete for the role of patient. The ambiguous noun is homonymous in its ACC.PL. and INST.PL. form. It is initially interpreted as the patient depending on the verb (ACC.PL.), but later, when the actual patient is encountered, needs to be reanalyzed as an agent depending on the verbal adjective (INST.PL.).

(23) Robert zvědavě vyhledal
Robert curiously seeked_out
student-y/student-em zmiňované oddělen-í
student-ACC.M.PL./INST.M.PL/student-INST.M.SG mentioned department
v muzeu.
-ACC.N.SGin museum.
'Robert curiously seeked out the museum department mentioned by the students/the student.'

Question: Vyhledal Robert studenty? (*Did Robert seek out the students?*)

AmbMis: Robert zvědavě vyhledal studenty. (*Robert curiously seeked out the students.*)

DisCor: Robert zvědavě vyhledal oddělení v muzeu. (*Robert curiously seeked out the museum department.*)

AmbCor: Robert zvědavě vyhledal studentem zmiňované oddělení v muzeu. (*Robert curiously seeked out the museum department mentioned by the student.*)

Type VIII: deverbsubj

This type is similar to Type VI and Type VII, except here, the two nouns compete for the role of agent. The ambiguous noun is homonymous in its NOM.PL. and INST.PL. form. It is initially interpreted as the agent depending on the verb (NOM.PL.), but later, when the actual agent is encountered, needs to be reanalyzed as an agent depending on a verbal adjective (INST.PL.).

(24)

Na	festival	dorazili	herc-i/herc-em	obdivovaní
At	festival	arrived	actor-NOM.M.PL./INST.M.PL/actor-INST.M.SG	admired

režiséř-i	ze	zahraničí.	
directors	-ACC.N.SG	from	abroad.

'The directors from abroad admired by the actors/the actor arrived at the festival.'

Question: Dorazili na festival herci? (*Did actors arrive at the festival?*)

AMBMIS: Robert zvědavě vyhledal studenty. (*Robert curiously seeked out the students.*)

DISCOR: Robert zvědavě vyhledal oddělení v muzeu. (*Robert curiously seeked out the museum department.*)

AMBCOR: Robert zvědavě vyhledal studentem zmiňované oddělení v muzeu. (*Robert curiously seeked out the museum department mentioned by the student.*)

Type IX: adjective

This type is similar to types VI, VII and VIII, however it does not use a verbal adjective but a regular adjective on which the ambiguous noun depends (e.g. *close to...* instead of *mentioned by...*. The ambiguous and the disambiguating noun compete for the position of an indirect object depending on the verb. The ambiguous noun is homonymous in its LOC.SG. and DAT.SG. form. It is initially interpreted as an indirect object (LOC.SG.), but when another noun, which can only be identified as the indirect object, is encountered, it needs to be reanalyzed as an argument of the adjective (DAT.SG.).

(25) Matěj diskutoval o firm-ě/firm-ám
Matěj argued about company-LOC.F.SG./DAT.F.SG./company-DAT.F.PL.
nedostupných peněz-ích z projektu.
unavailable money-LOC.F.PL. from project.
'Matěj argued about the project money unavailable to the company/the companies.'

Question: Diskutoval Matěj o firmě? (*Did Matěj argue about the company?*)

AmbMis: Matěj diskutoval o firmě. (*Matěj argued about the company.*)

DisCor: Matěj diskutoval o nedostupných penězích z projektu. (*Matěj argued about the unavailable project money.*)

AmbCor: Matěj diskutoval o firmám nedostupných penězích z projektu. (*Matěj argued about the project money unavailable to the companies.*)

Type X: coordovssubj

This type is very similar to one of the English garden-path structures used by, for example, Christianson and Luke (2011). Here, a conjunction *and* is initially interpreted as connecting two objects of a single verb but needs to be reanalyzed as connecting two clauses when another verb, which would otherwise lack an object, is encountered. Similarly, the ambiguous noun, which follows after the conjuction, is initially interpreted as an object of the first verb (ACC.SG.) but has to be reanalyzed as an object of the second verb. The second sentence had an OVS word order.

(26) Míša uklidila kádink-u a/,ale zkumavk-u
Míša put_away beaker-ACC.M.SG. and/but test_tube-ACC.M.SG/ACC.M.SG
údajně odnesla Lída.
allegedly carried_away Lída.
'Míša put away the beaker and/but Lída allegedly carried away the test tube.'

Question: Uklidila Míša zkumavku? (*Did Míša put away the test tube?*)

AmbMis: Míša uklidila zkumavku. (*Míša put away the test tube.*)

DisCor: Lída údajně odnesla zkumavku. (*Lída allegedly carried away the test tube.*)

AmbCor: Míša uklidila kádinku, ale zkumavku údajně odnesla Lída. (*Míša put away the beaker, but Lída allegedly carried away the test tube.*)

Type XI: coordsvo

This type is similar to type XI but the ambiguous noun, which follows after the conjunction, has slightly different properties. It is initially interpreted as an object of the first verb but later needs to be reanalyzed as a subject of the second verb. The second clause has an SVO word order.

(27) Pavel přeinstaloval ovladač-0 a/,ale program-0
Pavel reinstalled driver-ACC.M.SG. and/but program-ACC.M.SG/NOM.M.SG
pořád hlásil chybu.
still reported error.
'Pavel reinstalled the driver and/but the program still reported an error.'

Question: Přeinstaloval Pavel program? (*Did Pavel reinstall the program?*)

AmbMis: Pavel přeinstaloval program. (*Pavel reinstalled the program.*)

DisCor: Program pořád hlásil chybu. (*The program still reported an error.*)

AmbCor: Pavel přeinstaloval ovladač, ale program pořád hlásil chybu. (*Pavel reinstalled the driver, but the program still reported an error.*)

Appendix B

Mean response accuracies and RTs for different types of garden-paths

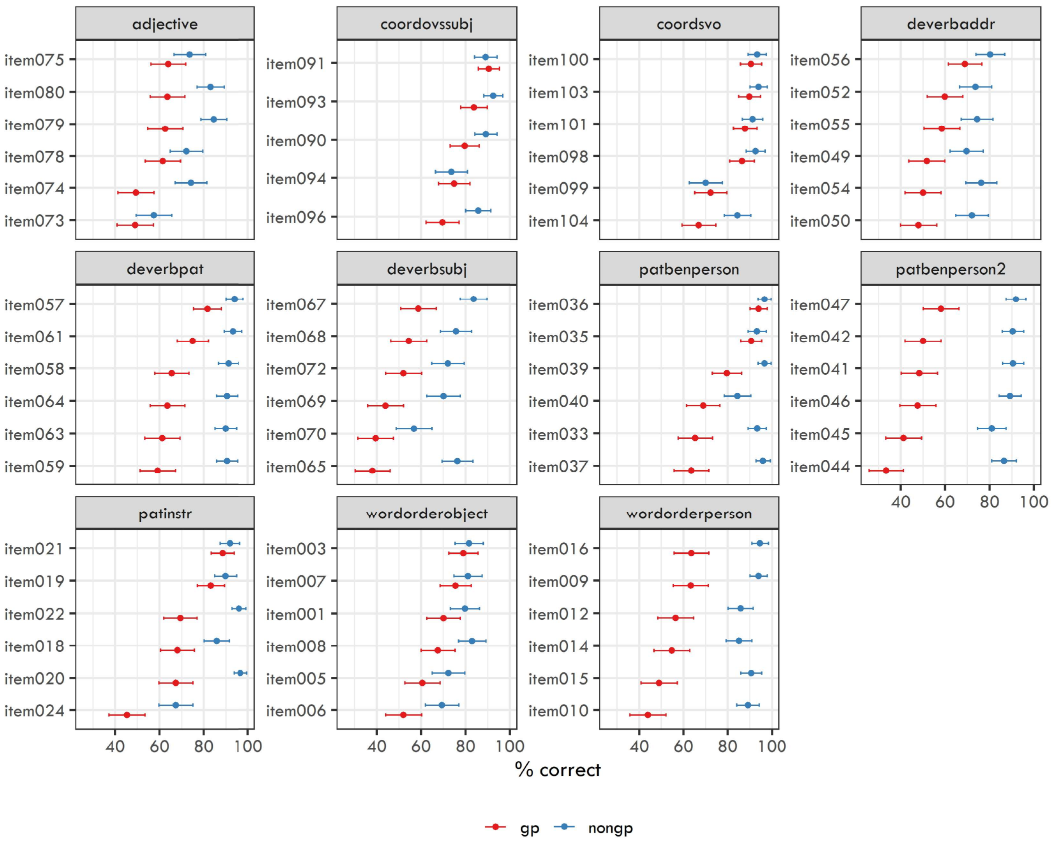

Figure B1

Mean response accuracies for each item in the two conditions (garden-path and non-garden path) depending on the type of construction. The error bars represent 95% confidence intervals of the means. Items are sorted in descending order based on their mean accuracy for garden-path conditions.

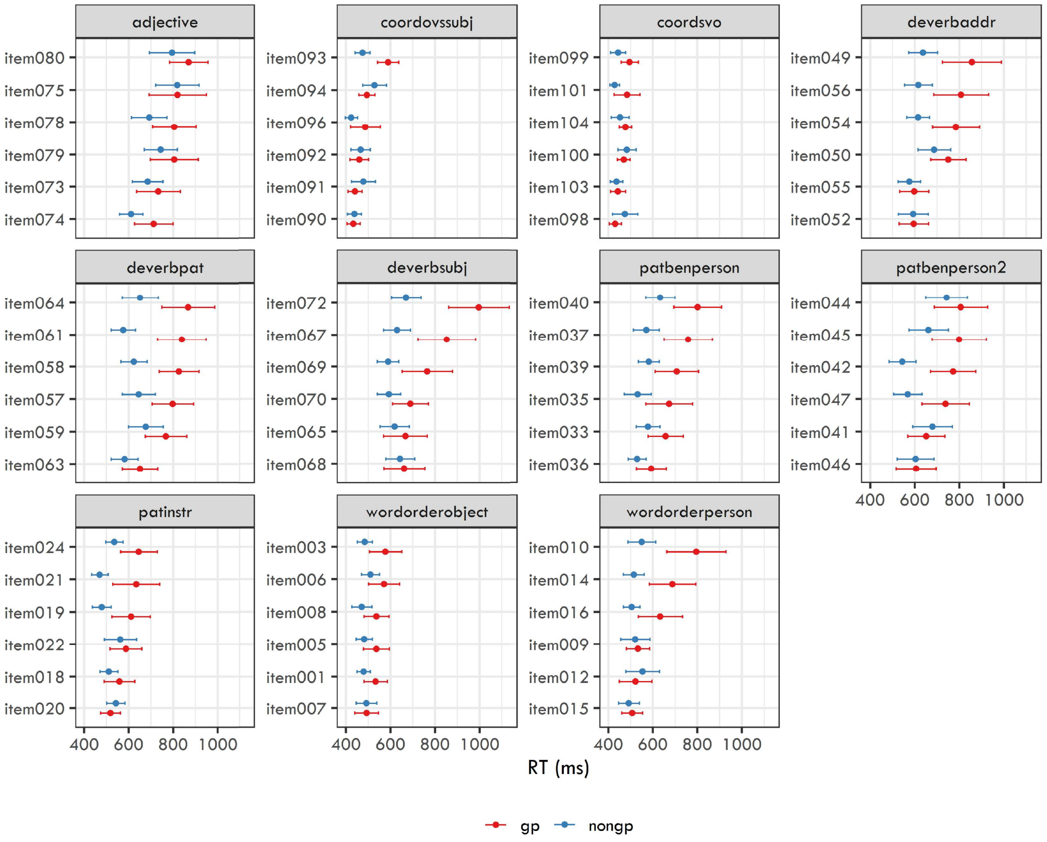

Figure B2

Mean reaction times on the disambiguating region for each item in the two conditions (garden-path and non-garden path) depending on the type of construction. The error bars represent 95% confidence intervals of the means. Items are sorted in descending order based on their mean reaction times for garden-path conditions.

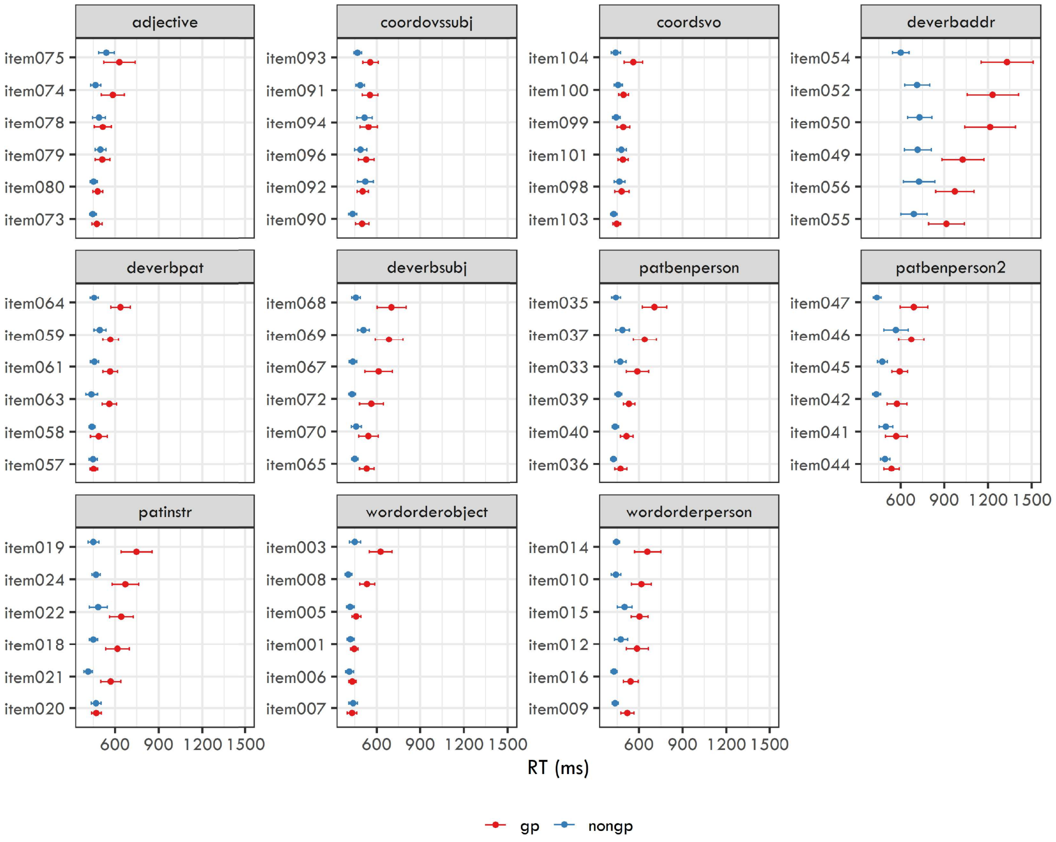

Figure B3

Mean reaction times on the spillover region for each item in the two conditions (garden-path and non-garden path) depending on the type of construction. The error bars represent 95% confidence intervals of the means. Items are sorted in descending order based on their mean reaction times for garden-path conditions.
